# Static Magnetic Field Stimulation Enhances Oligodendrocyte Differentiation and Secretion of Neurotrophic Factors

**DOI:** 10.1038/s41598-017-06331-8

**Published:** 2017-07-27

**Authors:** Ankshita Prasad, Daniel B. Loong Teh, Agata Blasiak, Chou Chai, Yang Wu, Payam M. Gharibani, In Hong Yang, Thang T. Phan, Kah Leong Lim, Hyunsoo Yang, Xiaogang Liu, Angelo H. All

**Affiliations:** 1 Department of Biomedical Engineering, National University of Singapore, E4, 4 Engineering Drive 3, Singapore, 117583 Singapore; 2 Singapore Institute of Neurotechnology (SINAPSE), National University of Singapore, 28 Medical Drive, 5-COR, Singapore, 117456 Singapore; 3 National Neuroscience Institute, 11 Jalan Tan Tock Seng, Singapore, 308433 Singapore; 4 Department of Electrical and Computer Engineering, National University of Singapore, 4 Engineering Drive 3, Singapore, 117583 Singapore; 5 Department of Biomedical Engineering, John Hopkins School of Medicine, 701C Rutland Avenue 720, Baltimore, MD 21205 USA; 6 Department of Surgery, Yong Loo Lin School of Medicine, National University of Singapore, Singapore, 119228 Singapore; 7 Department of Physiology, 2 Medical Drive, MD9, National University of Singapore, 117593 Singapore, Singapore; 8 Duke-NUS Medical School. 8 College Road, 169857 Singapore, Singapore; 9 Department of Chemistry, National University of Singapore, 3 Science Drive 3, Singapore, 117543 Singapore; 10 Department of Neurology, John Hopkins School of Medicine, 701C Rutland Avenue 720, Baltimore, MD 21205 USA

## Abstract

The cellular-level effects of low/high frequency oscillating magnetic field on excitable cells such as neurons are well established. In contrast, the effects of a homogeneous, static magnetic field (SMF) on Central Nervous System (CNS) glial cells are less investigated. Here, we have developed an *in vitro* SMF stimulation set-up to investigate the genomic effects of SMF exposure on oligodendrocyte differentiation and neurotrophic factors secretion. Human oligodendrocytes precursor cells (OPCs) were stimulated with moderate intensity SMF (0.3 T) for a period of two weeks (two hours/day). The differential gene expression of cell activity marker (c-fos), early OPC (Olig1, Olig2. Sox10), and mature oligodendrocyte markers (CNP, MBP) were quantified. The enhanced myelination capacity of the SMF stimulated oligodendrocytes was validated in a dorsal root ganglion microfluidics chamber platform. Additionally, the effects of SMF on the gene expression and secretion of neurotrophic factors- BDNF and NT3 was quantified. We also report that SMF stimulation increases the intracellular calcium influx in OPCs as well as the gene expression of L-type channel subunits-CaV1.2 and CaV1.3. Our findings emphasize the ability of glial cells such as OPCs to positively respond to moderate intensity SMF stimulation by exhibiting enhanced differentiation, functionality as well as neurotrophic factor release.

## Introduction

Static magnetic fields (SMF) are constant magnetic fields that do not vary in intensity or direction over time and have a frequency of 0 Hz. Permanent magnets or electromagnetic coils with direct current are the most common sources of SMF. In the last decade, accumulating evidence have established the ability of biological systems to detect and respond to a wide range of magnetic fields such as static and oscillating magnetic fields^1–3^. Investigations dating as early as 1970s have shown that the central nervous system (CNS), in particular, is highly sensitive and responsive to magnetic fields^4, 5^. More recent studies have documented the effects of magnetic field on neurogenesis^6, 7^, neuroprotection^8^, synaptic plasticity and remodelling^9^, behavior, memory and cognitive function^10^ as well as differentiation of neural stem cells^11, 12^. Surveying the available literature in this field, it is observed that most of these studies focus on the cellular-level effects of low/high frequency oscillating magnetic field such as those used in Transcranial Magnetic Stimulation. These fields inherently carry an associated electric field that can induce electrical and chemical changes in excitable cells such as neurons. In contrast, the effects of a homogenous SMF on non-excitable CNS cells such as glial cells are less investigated. Thus, the exact molecular mechanisms and signal transduction initiated by SMF on non-excitable cells remains to be elucidated. Rosen *et al*. proposed a model explaining the cellular level effects of SMF^13^. They hypothesized that SMF causes a magnetic reorientation of membrane phospholipids and the ion channels embedded in them by diamagnetic anisotropy effects. The evidence in favor of this model can be found in various other studies that demonstrate alteration of membrane properties, charge, redox potential and channel kinetics^14–16^. Specifically, changes in voltage operated calcium channel (VOCC) activity, alteration in intracellular calcium flux and membrane depolarization have been the most consistent effect observed under SMF stimulation, as reviewed by Pall *et al*.^3^.

Oligodendrocytes (OLs) are glial cells that play the critical role of myelinating and insulating axons to maintain saltatory conduction in the CNS. Reports indicate that myelination is regulated by ion channel activation^17^ or neuronal activity^18, 19^. The axonal electrical activity is known to increase OPCs maturation and myelination through axon-derived neurotransmitters: ATP, glutamate, adenosine and GABA^20^. A majority of these synaptic inputs induces cellular depolarization in OPCs and affects the calcium signaling cascades^17, 21^. In the absence of detectable output signals from OPCs, it can be hypothesized that the electrical signals induced membrane depolarization may have a cell intrinsic role such as regulating the development of OPCs and their maturation from precursors to myelinating oligodendrocytes.

In this study, we investigated the effects of moderate intensity (0.3 T) SMF over two weeks (2 hours/day continuous stimulation) on human oligodendrocyte precursors and report an increase in gene expression of cell activity marker (*c*-*fos*), pre-myelinating OL marker (*CNP*) and mature OL marker (*MBP*). We tested the functionality of SMF stimulated OLs in Dorsal Root Ganglion (DRG) microfluidics platform and observed a significant increase in the number of myelinating cells and a decrease in the number of nude axons. Additionally, we have interestingly noticed that SMF stimulation significantly increases the gene expression and secretion of neurotrophic factors- Brain Derived Neurotrophic Factor (*BDNF*) and Neurotrophin 3 (*NT3*). We also report that SMF stimulation increases the intracellular calcium influx in OPCs as well as the gene expression of L-type channel subunits: CaV1.2 and CaV1.3. Overall, our results suggest that SMF stimulation promotes differentiation of OPCs and enhances their myelination potential as well as increases the secretion of neurotrophic factors BDNF and NT3.

## Results and Discussion

### Design of the set-up for Static Magnetic Field (SMF) stimulation

**Figure 1:**
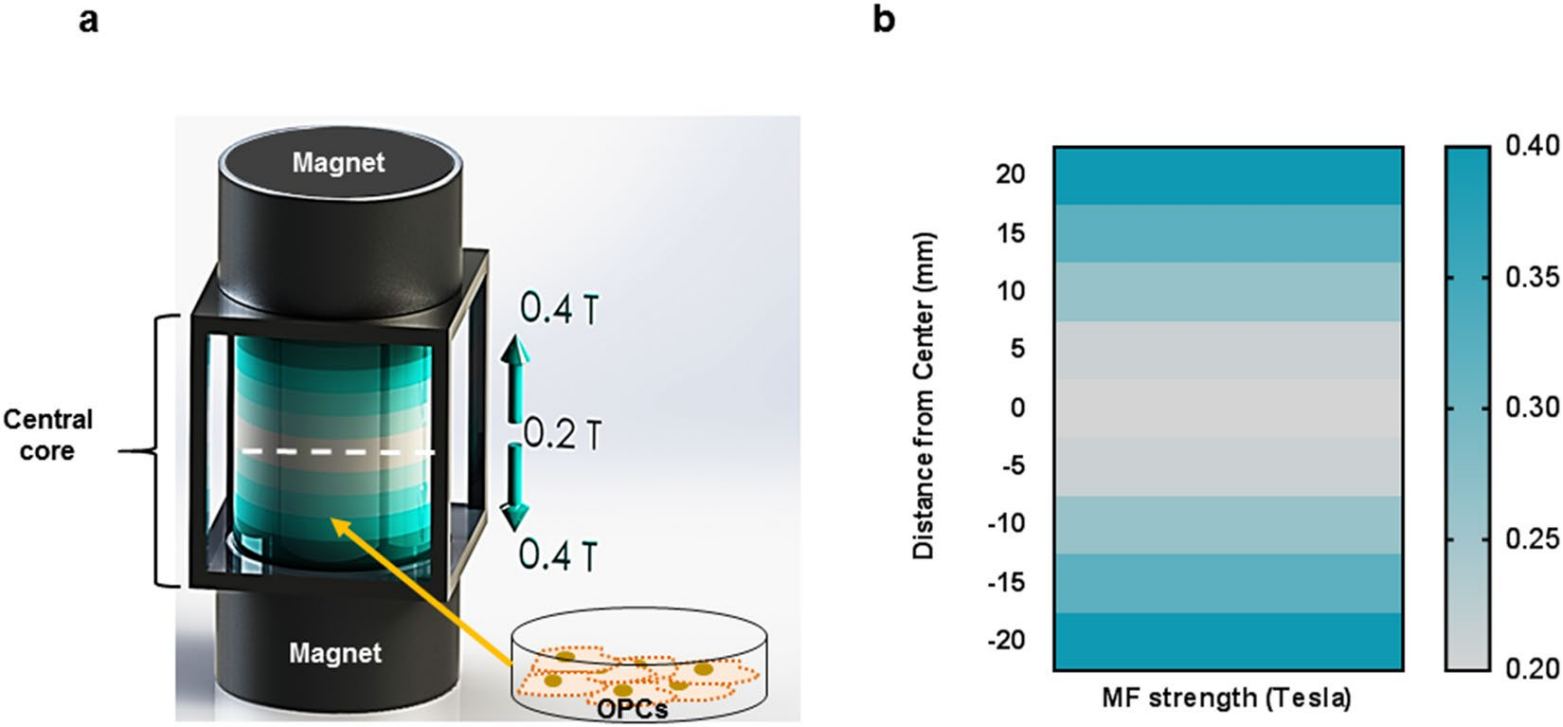
Schematic Illustration of the SMF stimulation set-up. (**a**) Two parallel magnets with opposite polarity produce a uniform static magnetic field in the central core. The magnetic induction range represented by the shaded blue columns. The field intensity varies from 0.2 T at the center line (white dashed) to 0.4 T at the base of the two magnets. The OPCs are placed at a distance of 1 cm from the base to expose them to 0.3 T magnetic field. (**b**) The characterisation of the magnetic field strength with respect to the vertical distance from the center line towards the base of the two magnets.

The SMF stimulation system was designed in accordance to the following parameters^16^: (i) Moderate intensity of static magnetic field (0.1–1 T), (ii) Unidirectional field with no reverse field passing through sample, and (iii) homogenous, uniform magnetic field. Static magnetic field was generated by placing two circular NdFeb magnets with opposite polarities parallel to each other as shown in Fig. 1a and described in the Material and Methods section. The magnetic induction range of the set-up was 0.2–0.4 T Fig. 1b shows the magnetic field intensity as measured at intervals of 5 mm from the center line of the device. The magnetic field strength is minimum (0.2 T) at the center (x = 0) and symmetrically increases as the distance approaches towards the two magnets on the top and bottom. Negligible variation (<1 mT) of magnetic flux was observed in the horizontal plane indicating an absence of gradient magnetic field. Hence, the cells are placed in the central core at a distance of 1 cm from the base to expose them to 0.3 T static magnetic field intensity as shown in Fig. 1a.

### Characterisation of human derived oligodendrocyte precursor cells

**Figure 2:**
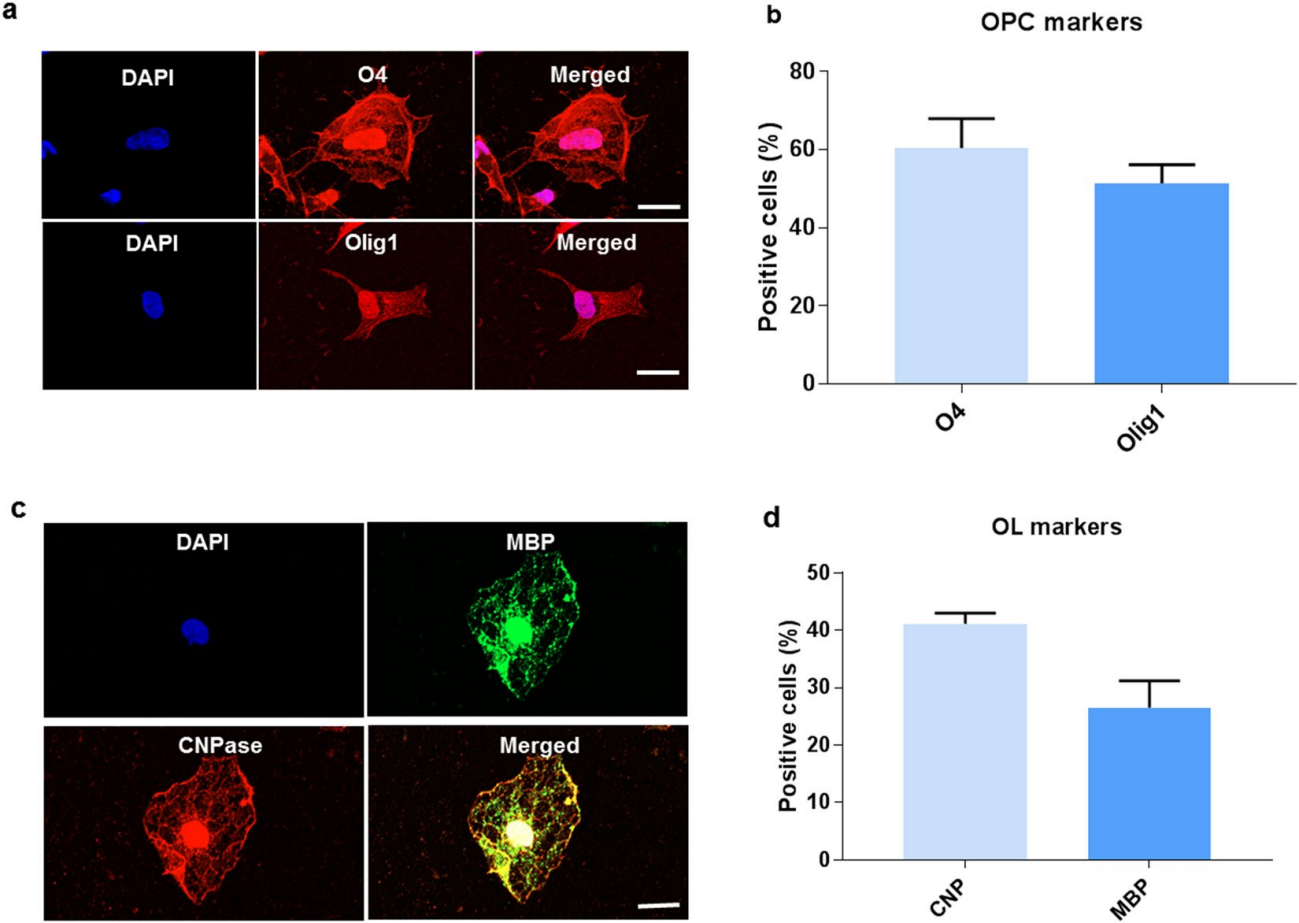
Characterisation of the oligodendrocyte precursor cells. (**a**) Representative immunofluorescence images of OPCs stained for O4 and Olig 1, (**b**) Estimate of the percentage of cells staining positive for OPC markers. (**c**) Representative immunofluorescence images of mature OLs stained for CNP and MBP, (**d**) Estimate of the percentage of cells staining positive for OL markers. Data represented as mean ± SEM. Scale bar = 2 µm.

**Figure 3:**
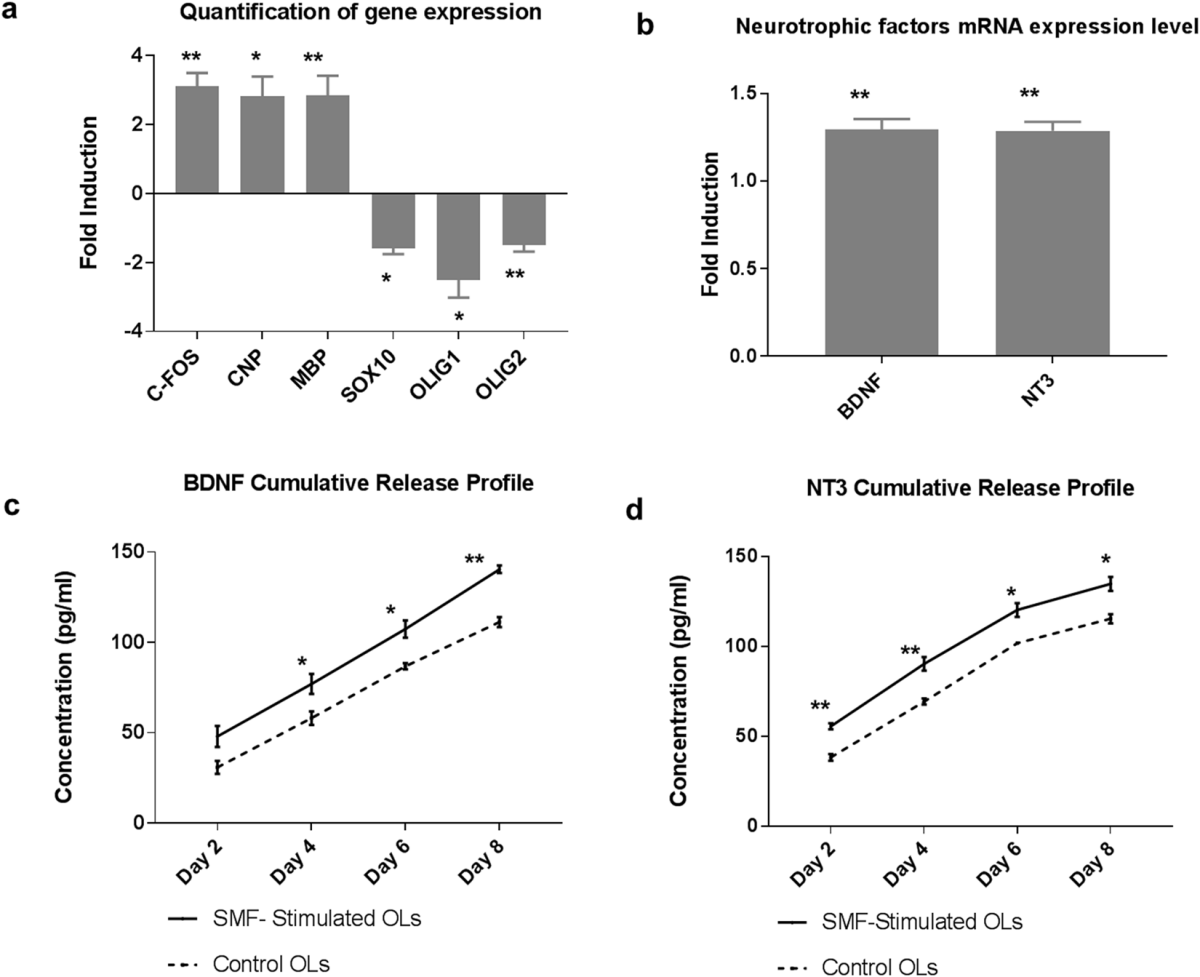
Effects of SMF on oligodendrocyte differentiation and neurotrophic factor release. (**a**) Quantification of gene fold changes in SMF stimulated oligodendrocytes as compared to control (non-stimulated), (**b**) mRNA expression levels of neurotrophins BDNF and NT3 in SMF stimulated oligodendrocytes as compared to control, (**c**,**d**) Cumulative release profile of BDNF and NT3 (*p < 0.05, **p < 0.01) in SMF stimulated and non-stimulated cells.

The genomic profile of differentiating oligodendrocytes indicates that OPCs express the O4 antigen^22, 23^. We sorted O4^+^ cells in order to derive a homogenous population of OPCs. Next, we tested their immunoreactivity for O4 as well as Olig 1 which is also a marker of early OPCs. Immunofluorescence characterisation of OPCs show 80.9% ± 4.7 of cells to be positive for O4 and 67.4% ± 5.1 for early OPC marker Olig 1, as reported in Fig. 2b. The cells were then cultured for 2 weeks in oligodendrocyte differentiation medium with T3 in the absence of mitogens to validate their ability to differentiate into mature oligodendrocytes (Fig. 3b). 74.9% ± 5.6 of cells are seen to be positive for pre-myelinating OL marker CNP and 47.4% ± 3.1 of cells were positive for MBP, which is a marker for mature, fully differentiated oligodendrocytes^23^. Representative immunofluorescence images are provided in Figs 2 and 3a.

### Quantification of genomic changes

To verify our hypothesis that SMF is able to stimulate OPCs, we measured the gene expression of *c*-*fos*, which is an indicator of cellular activity. The result (Fig. 3a) showed a 3.1 ± 0.54 fold increase in *c*-*fos* gene expression, which is a significant enhancement (p = 0.0048, n = 4). *c*-*fos* is an immediate-early gene belonging to the activator protein-1 (AP-1) transcription factor family that is widely used as a marker of neuronal activity^24^. *c*-*fos* is also well-established cellular activity marker which is known to regulate cell proliferation and differentiation. It is known to be induced by a range of stimuli such as electrical excitation, calcium influx and membrane depolarization^25, 26^. Interestingly, the documented effects of magnetic field on *c*-*fos* gene expression are found to be contradictory and largely dependent on stimulation parameters^27–30^. Our results indicate that 2 hours/day of SMF stimulation (0.3 T) for a period of 14 days, significantly enhances cellular activity of OPCs as indicated by a significant increase in *c*-*fos* gene expression. With an aim to investigate the effects of SMF on human OPC differentiation, the differential gene expression was measured for selected genes as shown in Fig. 3a. The result indicates a statistically significant (p = 0.03), 2.8 ± 0.8 fold increase in *CNP* expression which is a marker of pre-myelinating OLs. Additionally, an increase of 2.8 ± 0.8 fold in *MBP* expression, which is a marker for mature myelinating oligodendrocytes (p = 0.002) was detected. *CNP* is a cytoplasmic peripheral membrane protein that forms approximately 4% of total myelin protein in the CNS^31^. *CNP* expression is found to be highly up-regulated in late phase OPCs^23, 32^, which plays a critical role in process extension and cytoskeleton remodeling of OPCs as well as maintenance of the myelin sheath^33^. *MBP*, located at the cytoplasmic surface of myelin membranes, is a basic membrane-associated adhesive protein that is critical for myelination of axons^34^. *MBP* expression is found specifically in mature OLs that are capable of wrapping around axons and initiating myelination^35^. We also observed a significant decrease in early OPC markers such as *Olig1* (2.5 fold ± 0.73, p = 0.01), *Olig2* (1.5 fold ± 0.27, p = 0.002) and *Sox10* (1.58 fold ± 0.25, p = 0.03). *Olig1* and *Olig2* are robustly expressed by immature OPCs in the CNS, but their expression is reported to be down-regulated in mature OLs^36^. Overall, these results suggest a down regulation of OPC markers and an up-regulation of mature OL markers indicating an enhancement of their differentiation process.

### Quantification of SMF effects on Cell Proliferation

**Figure 4:**
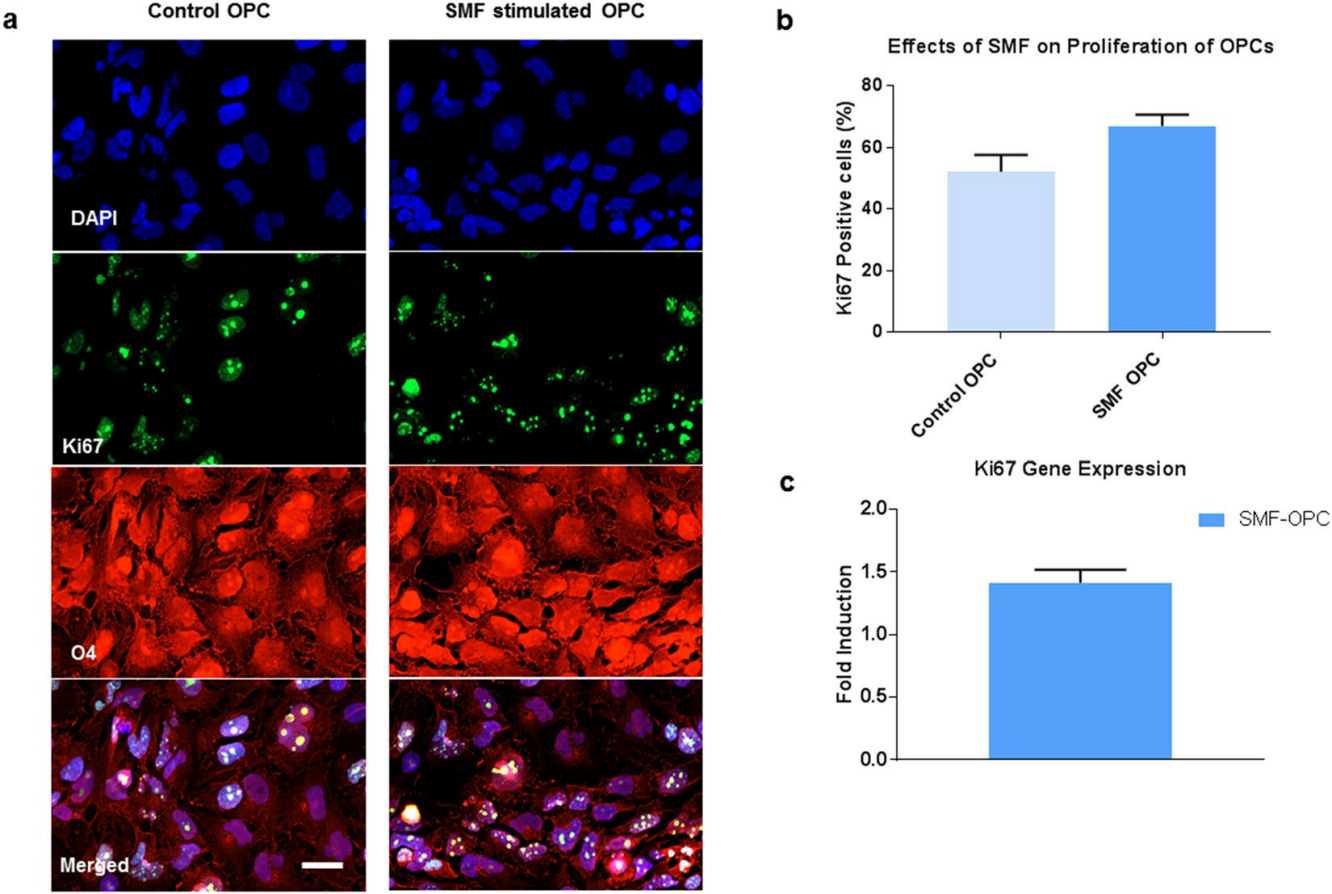
Effects of SMF on OPC proliferation. (**a**) Representative images of OPC stained with O4 and Ki67 markers, (**b**) Quantification of Ki67 positive cells in SMF stimulated OPCs and control, (**c**) mRNA expression level of proliferative marker Ki67 in SMF stimulated OPCs as compared to control OPCs. Data represented as mean ± SEM. Scale bar = 20 µm.

The effects of SMF stimulation on the proliferation rate of OPCs was measured using the Ki67 proliferation marker. The immunofluorescence results (Fig. 4b) indicate that 52% ± 5 of control OPCs and 67% ± 4 of SMF stimulated OPCs were positive for Ki67. However, this increase in proliferating cells were not statistically significant (p = 0.08). The gene expression levels of Ki67 was also validated using qRT-PCR. As shown in Fig. 4c, although Ki67 is up-regulated in SMF stimulated OPCs (1.4 ± 0.1 fold), the difference was not found to be statistically significant (p = 0.3). Previous reports have also indicated that SMF stimulation either reduces or has no effect on cell proliferation^37–39^.

### Quantification of secretion of neurotrophic factors

The RNA analysis of SMF stimulated and non-stimulated OPCs, differentiating to mature OLs, indicates a 1.3 fold increase in the gene expression of neurotrophic factors BDNF and NT3, (p = 0.003, n = 3) as shown in Fig. 3b. To verify the increase in protein secretion of these neurotrophic factors, ELISA assays were performed and cell-culture supernatants were collected every 48 hours. Figure 3c and d show the cumulative release profile of the neurotrophic factors (SMF stimulated and non-stimulated OPCs) over a period of 8 days. This period was sufficient to obtain a release profile of the targeted neurotrophic factors. On day 2 post SMF stimulation, the BDNF release from SMF stimulated OPCs was 48 ± 5. 8 pg/ml as compared to 30.9 ± 3.5 pg/ml for non-stimulated OPCs. Though this increase was not statistically significant, from day 4 onwards, a steady and statistically significant increase in the release of BDNF was observed. Similarly, the cumulative release profile of NT3 indicated a statistically significant increase in the amount of NT3 secretion by the stimulated OPCs when compared to non-stimulated OPCs. Table 1 provides a summary of the release profile of BDNF and NT3 along with the statistical information.

**Table 1:** Summary of cumulative release profile of BDNF and NT3 in SMF stimulated and control OPCs.

Time-point	BDNF cumulative release		*p*-value	NT3 cumulative release		*p*-value
	Stimulated (pg/ml)	Control (pg/ml)		Stimulated (pg/ml)	Control (pg/ml)	
Day 2	48.0 ± 5.8	30.9 ± 3.5	2.500	55.6 ± 1.7	38.3 ± 1.9	0.002
Day 4	75.6 ± 6.2	58.1 ± 3.7	0.047	90.3 ± 3.7	69.3 ± 1.7	0.007
Day 6	107.4 ± 4.7	86.86 ± 1.7	0.010	120.3 ± 2.1	95.23 ± 3.6	0.040
Day 8	142.4 ± 2.0	111.3 ± 2.7	0.001	134.8 ± 3.8	115.4 ± 2.5	0.010

Date is represented as mean ± SEM.

Our results are consistent with the effects of pulsed magnetic field increasing neurotrophic factor release in the case of stimulating Schwann cells^40^ as well as high-frequency transcranial magnetic stimulation^41–43^. Although the exact molecular mechanism of SMF induced enhancement of neurotrophic factor release is yet to be elucidated, membrane depolarization and changes in calcium influx have been considered to be the underlying mechanism^44, 45^. Increasing the secretion of neurotrophic factors is significant in view of clinical applications and translational medicine. Most traumatic injuries create a hostile microenvironment that prevents cell survival and integration, thereby, inhibiting regenerative processes post-injury. Specifically, BDNF and NT3 are critical neurotrophic factors that provide tropic support to the neurons, promote neurogenesis, neuroprotection, myelination, endogenous stem cell proliferation and migration as well as modulate the microenvironment to enhance functional recovery^46–48^. Genetically modifying the transplanted cells to release neurotrophic factors has been one of the techniques to deliver neurotrophic factors to the endogenous microenvironment in the CNS^49–51^. However, low transfection efficiency and transfection induced cytotoxicity have limited the use of these approaches with reprogrammed cell lines. Therefore, it is noteworthy that SMF stimulation could enhance secretion of neurotrophic factors- BDNF and NT3 without the use of transgenes or modification of the cell’s genome.

### Quantification of intracellular calcium levels

**Figure 5:**
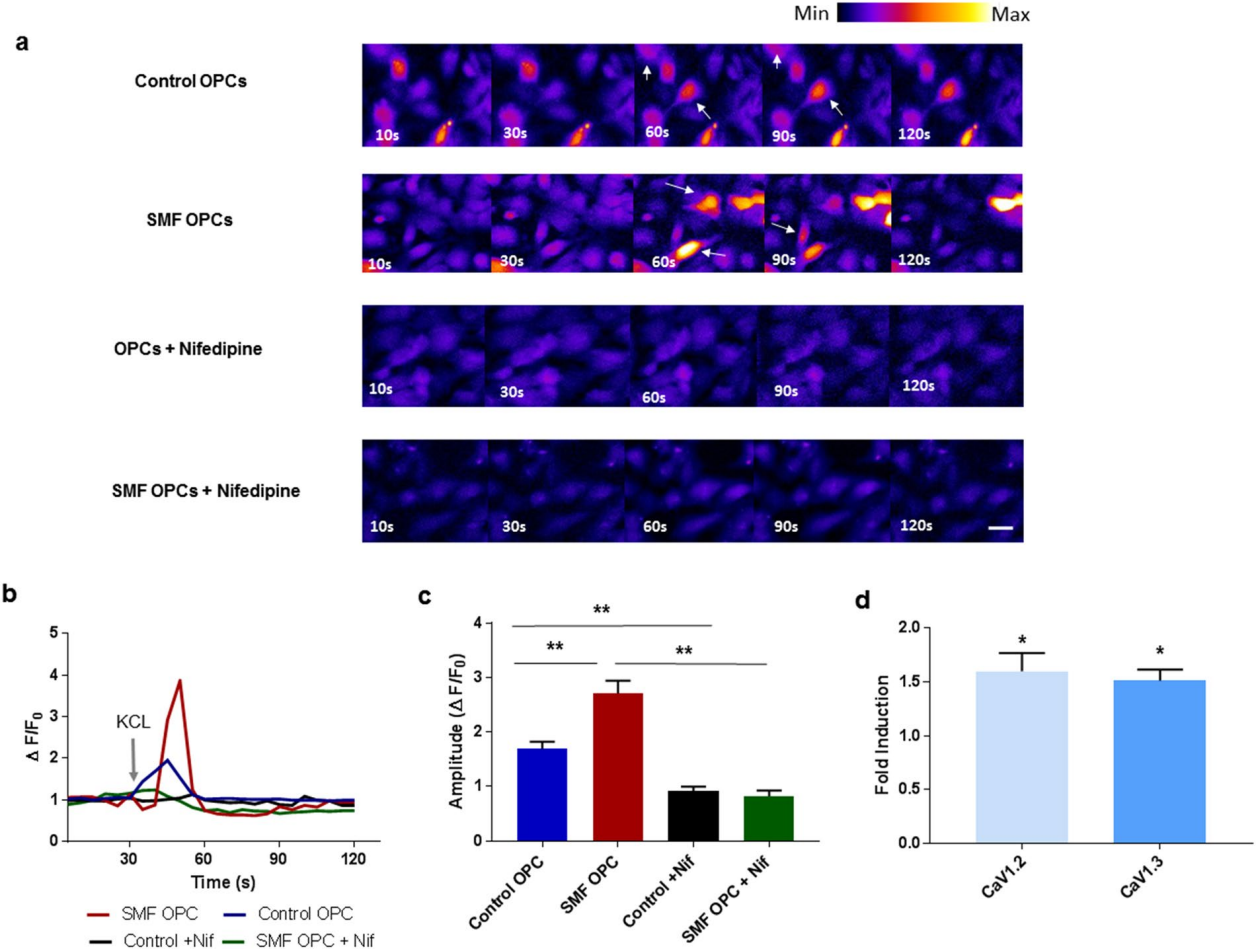
SMF effects on intracellular calcium levels and L-type channel subunits. (**a**) Representative scans from the time-lapse calcium imaging experiments showing some responding cells (white arrows) post KCL stimulation at 30 seconds, (**b**) Representative calcium response traces, grey arrow represents the KCL stimulation at 30 sec (**c**) Summary data showing significantly higher calcium transient amplitudes in SMF stimulated OPCs (n = 12 cells), (**d**) Quantification of mRNA expression levels of CaV1.2 and CaV1.3 in SMF stimulated OPCs as compared to control OPCs. Scale bar = 20 µm. *p < 0.05, **p < 0.01. Error bars indicate SEM.

Although the exact molecular mechanism of SMF mediated modulation of cellular function is not known, several reports document an increase in intracellular calcium levels in diverse cell types post SMF exposure^52–56^. Also, theoretical models predict that SMF alters the biophysical properties of the cell membrane and consequently changes the kinetics of the embedded channels^13–15^. We investigated the effects of SMF stimulation on the intracellular calcium levels in OPCs using Fluo-4 AM, a membrane-permeant calcium probe. Time-lapse imaging of Fluo-4-AM dye-loaded OPCs were performed after exposing the OPCs to SMF for 2 weeks (2 hours/day). Figure 5c indicates that SMF stimulated OPCs have a significantly higher calcium transient amplitude (post KCL stimulus) when compared to the control non-stimulated OPCs (ΔF_amplitude_ for SMF-OPC is 2.70 ± 0.23 and ΔF_amplitude_ for Control OPCs is 1.69 ± 0.13; p = 9.0 × 10^−6^). To investigate if this increase in cytosolic calcium level after KCL induced membrane depolarization was mediated by L-type calcium channels, the OPCs were treated with L-type channel blocker Nifedipine for 15 minutes before being imaged. The calcium transient amplitude value for Nifedipine treated SMF-stimulated and control OPCs were significantly decreased post treatment with Nifedipine (ΔF_amplitude_ = 0.83 ± 0.10 for Nifedipine treated SMF OPCs, ΔF_amplitude_ = 0.92 ± 0.07 for Nifedipine treated control OPCs; p < 0.0001 and p = 0.003) when compared to the SMF OPC and control OPCs respectively.

These results suggest that the OPCs stimulated with SMF have a higher permeability to calcium ions, as deduced from the higher calcium influx. Although lacking conclusive empirical evidence, reports indicate an alteration of channel kinetics L-type Voltage Operated Calcium Channel (VOCC) and membrane fluidity to possibly be the cause of higher calcium influx^3^. Since L-type calcium channels are reported to play an important role in OPC differentiation^57^, we further investigated if SMF stimulation alters the gene expression of L-type VOCCs. The α1 subunit of the L-type VOCC is encoded by four different subtypes known to as CaV1.1, CaV1.2, CaV1.3, and CaV1.4. Amongst these only CaV1.2 and CaVO1.3 are expressed in the brain^58^ as well as cultured OPCs^57^. Figure 5d shows the statistically significant 1.6 ± 0.2 (p = 0.02) fold up-regulation of CaV1.2 gene and 1.5 ± 0.1 (p = 0.03) fold up-regulation of CaV1.3 gene in OPCS that were exposed to SMF stimulation as compared to non-stimulated OPCs.

Several studies have established the importance of calcium signaling in OPC differentiation, maturation, myelination capacity as well as migration^59, 60^. Interestingly, the RNA-sequencing transcriptome database reveals high expression of L-type calcium channel subunits in OPCs and down-regulation of L-type VOCCs in myelinating oligodendrocytes^61^. Cheli *et al*. also report that the expression of VOCCs in the oligodendroglial lineage is highly regulated, suggesting that there may be a precise and narrow time window in which VOCCs affect OPC differentiation and myelination^57^. Our results present the possibility that the increase in intracellular calcium levels post SMF exposure is related to an up-regulation of CaV1.2 and CaV1.3 calcium channel subunits. Although the exact molecular mechanism remains to be investigated, it is possible that the improvement in oligodendrocyte differentiation as well as neurotrophic factor release could be induced by these alterations in calcium levels post SMF stimulation.

### Functional assessment of stimulated OLs

**Figure 6:**
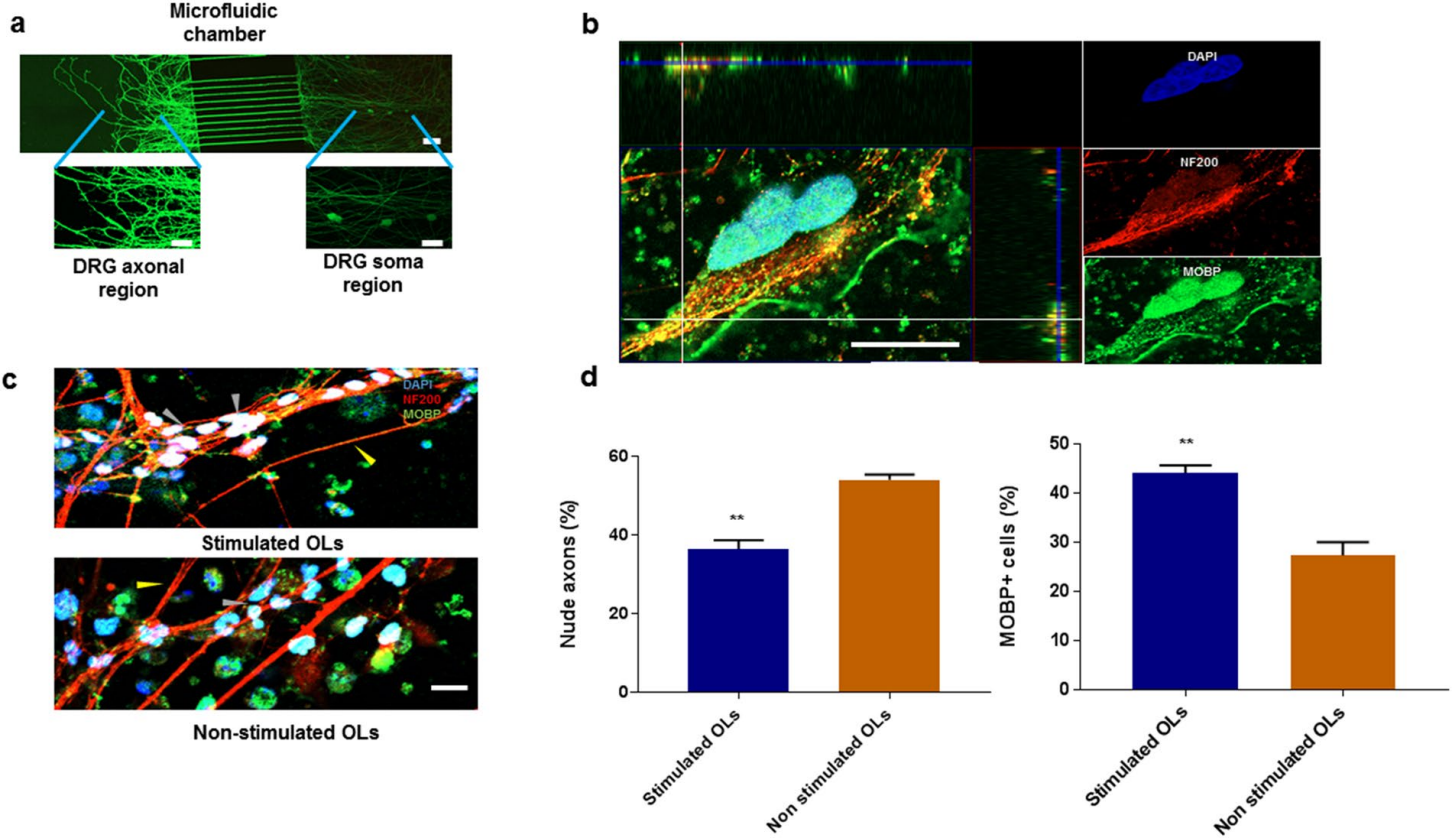
Investigation of myelination potential of SMF stimulated OPCs in DRG microfluidic chamber. (**a**) Illustration of microfluidic chamber with axonal and soma compartments connected with 10 μm wide and 500 μm long microchannels. DRG neurons (stained with Calcein) were plated in the somatic chamber and only axons (no cell body) extends to the axonal chamber. (**b**) Representative image demonstrating the wrapping of axons stained with Neurofilament-200 by MOBP+ oligodendrocytes. 3-D analysis of the z-stack images indicate co-localisation of myelinating cells (green) with axons (red) in the z-domain, (**c**) Representative image demonstrating an increase in myelinating cells (grey arrow) and decrease in nude axons (yellow arrow) in chambers containing SMF stimulated oligodendrocytes as compared to non-stimulated oligodendrocytes. The control OL chamber has fewer cells making contact with the axons. (**d**) Quantification of number of MOBP+ oligodendrocytes and nude axons. Scale bar = 20 µm, (**p < 0.01).

After having observed an increase in genes involved in differentiation and myelination, the effects of SMF stimulation on the functionality of these OLs were validated in an *in vitro* microfluidic chamber plated with DRG neurons. From our previous experience^62^, we noticed that OLs do not significantly express myelin associated proteins (e,g MBP, MAG, MOBP) in *in vitro* cultures devoid of myelinating substrates such as axons. Co-cultures of neurons with oligodendrocytes have been previously described^63–65^, but such systems do not allow the axon to extend sufficiently beyond the cell body and hence limit their exposure to the myelinating OLs. We therefore designed a PDMS microfluidic chamber consisting of two compartments; the cell body of the neurons is housed in the soma compartment, while the axons pass through the PDMS barrier and extend into the axonal compartment (Fig. 6a). The SMF stimulated OLs were placed in the axonal compartment in order to detect and quantify the myelination process. We define myelinating OLs (white arrows) as cells that are (i) positive for MOBP and (ii) co-localize with the axonal marker- Neurofilament 200 in the z domain. Nude axons were defined as axons that had more than 3/4th of their total surface area unwrapped with MOBP+ cells (yellow arrows). Figure 6b shows OLs, which are fully differentiated mature MOBP+ cells that co-localize with axons (stained with Neurofilament 200) and wrap around nude axons (indicated by z domain co-localization of MOBP+ cells with the axonal marker). Figure 6d reports a significant increase (p = 0.006, n = 3) in the percentage of myelinating MOBP + cells (44.2 ± 1.58%) in the axonal compartment that were plated with SMF stimulated OLs when compared to the non-stimulated OLs (27.4% ± 2.7). In addition, the compartment plated with SMF stimulated OLs had a lower percentage of nude axons (45.8% ± 1.3) when compared to the compartment that contained the control, non-stimulated OLs (63.5% ± 2) which is statistically significant (p = 0.002, n = 3). The exact molecular mechanism underlying this SMF stimulation mediated increase in the number of myelinating cells that co-localized with the axon is unclear. It is possible that SMF stimulation induces morphological changes and cytoskeleton remodeling in OLs that enhances cell migration leading to increased contact with the axons. Also, the genomic analysis of the SMF stimulated OLs shows an increase in cell maturation (up-regulation of CNP and MBP) which could be the underlying cause of an increase in number of myelinating cells and wrapped axons.

Various cell replacement strategies focus on transplantation of oligodendrocytes as a therapeutic approach to demyelinating diseases. It is critical for the transplanted oligodendrocytes to acquire maturation and functionality in order to be able to initiate myelination. In this study, we were able to enhance the maturation and myelination capacity of OPCs by stimulating them with a static magnetic field. This is a feasible and inexpensive approach that can be implemented in a diverse range of cell reprogramming techniques that aim at procuring functional oligodendrocytes for cell replacement strategies.

There are different doctrines and school of thoughts regarding the efficacy of SMF stimulation in clinical applications that has emerged in the recent years^66–68^. Studies by Olivierio *et al*.^66^ and Silbert *et al*.^67^ reported that transcranial static magnetic field stimulation (tSMS) could modulate the excitability of the motor cortex, but these results could not be replicated in a recent study by Kufner *et al*.^68^. In addition to SMF stimulation, there have been reports of electric field or oscillating field stimulation enhancing the endogenous differentiation of OPCs and promoting re-myelination post spinal cord injury^69, 70^. Our results indicate that *in vitro* moderate SMF stimulation enhances differentiation of oligodendrocytes and the secretion of neurotrophic factors without the necessity to generate any electrical field.

In summary, we report: (i) two hours/day for 14 days of Static Magnetic Field (0.3 T) stimulation influences OPCs to (ii) promote their differentiation into myelinating mature oligodendrocytes, (iii) enhances their myelination capacity and consequently their functionality, and (iv) promotes gene expression and secretion of neurotrophic factors- BDNF and NT3, as well as (v) increases the intracellular calcium influx and gene expression of CaV1.2 and CaV1.3 subunits. These findings emphasize the ability of glial cells such as OPCs to positively respond to moderate intensity SMF stimulation and presents the potential of manipulating OPC differentiation and myelination *in vivo* to design new therapeutic strategies for translational applications.

## Materials and Methods

### Static Magnetic Field stimulation system

Uniform Static Magnetic field was generated by placing two NdFeB magnetic disks (Grade N35, diameter of 5 cm) with opposite polarity parallel to each other (Fig. 1a). The magnets were mounted on an aluminum structure (6 cm × 6 cm × 6 cm) at a distance of 5 cm from each other to create uniform magnetic induction in the central cavity between the two magnets. Precise measurement of magnetic field strength was carried out using a digital teslamenter (FH52 Teslameter, Magnet Physics Inc.) to validate generation of a uniform field with minimum gradient. Oligodendrocyte Precursors (OPCs) were plated on Matrigel (Corning®) coated 35 mm cell culture dishes and placed inside the central cavity of the device at a height of 1 cm from the base to stimulate cells with 0.3 T magnetic field (Fig. 1a). The oligodendrocyte precursors were stimulated for two hours every day for a period of 14 days during their differentiation phase. Control OPCs (non-stimulated) were kept in an identical incubator where the ambient magnetic field was ~35 μT.

### Cell culture

The human OPCs used in this project was derived from induced pluripotent stem cells gifted by our collaborator Dr. Lim KL^71^. Somatic reprogramming of human amniotic cells was performed using a modification of the EBNA-1 based episomal reprogramming method described by Okita *et al*.^72^. The episomal vectors pCXLE-hOCT3/4-shp53-F, pCXLE-hSK, and pCXLE-hUL were purchased from Addgene (#27077, 27078, 27080, respectively). Actively growing amniotic cells was dissociated using 1x trypsin/EDTA solution and transfected with the three vectors using the Neon™ Transfection System 100 µL Kit (Life Technologies, MPK10096). The original protocol was modified to become feeder-independent in our laboratory by substituting mouse embryonic fibroblast feeder layer with Matrigel. Differentiation of oligodendrocyte from human iPS was performed according to the protocol described by Douvaras *et al*.^73^. After the cells attained the late oligodendrocyte progenitor stage, the O4 positive cells were sorted using MACS (Miltenyi Biotech) according to manufacturer’s protocol. The cells were plated on Matrigel (Corning®) coated T25 flasks and grown in OPC proliferating medium containing DMEM-F-12 (Gibco™) medium supplemented with EGF (Sigma Aldrich), PDGF-AA (Sigma Aldrich), B27 (Gibco™), Human Insulin (Gibco™) and anti-biotic/anti-mycotic solution (HyClone™). After achieving 80% confluency, the cells were dissociated using Accutase (StemPro®) and plated on 35 mm dish coated with Matrigel. After 24 hours the medium was changed to Oligodendrocyte (OL) differentiation medium containing DMEM-F12 supplemented with 40 ng/ml 3,3′,5 Triido-L-thyronine (T3). The cells were then placed inside the designed magnetic stimulator and cultured in a 37 °C incubator with 5.0% CO_2_.

### Molecular assessment

#### Immunofluorescence staining

The OPCs were grown on Matrigel coated four well plates (IbidiR Inc.) at a cell density of 20,000 cells. The OPCs were fixed with 4% paraformaldehyde (PFA) in PBS for 10 minutes at 4 °C. The fixative solution was removed and cells were washed three times with PBS for 5 min each at 4 °C. Cells were permeabilised and blocked with blocking buffer (PBS solution containing 0.2% (w/v) Triton X-100 (Sigma) and 1% Donkey serum (Sigma) for 30 minutes at 4 °C. Primary antibodies O4 (Millipore,1:200), MBP (Invitrogen,1:200), Olig1, CNP, Neurofilament 200, MOBP (All from Millipore, 1:500), Ki67 (1:200, Abcam) was diluted in blocking buffer and incubated overnight at 4 °C. Samples were then washed three times with washing buffer (PBS containing 1% Triton X-100) for 10 minutes each at 4 °C. Secondary antibodies were added (Alexa 546 anti-mouse, Alexa 488 anti-rabbit, Alexa 488-anti-mouse, Alexa 488 anti-chicken, Thermo Fisher Scientific, 1:200) to the washing buffer and incubated at room temperature for 4 hours. Cells were then washed three times with washing buffer for 5 minutes each at room temperature. Nuclei were stained using 4′,6-diamidino-2-phenylindole (DAPI, 1:500) solution (Sigma) for 10 min at room temperature. DAPI solution was aspirated and three rinses with washing buffer for 5 minutes each at room temperature was performed. Mounting medium DAKO was added and the samples were incubated at 4 °C overnight.

#### RNA extraction and cDNA synthesis

1 ml of Accutase (StemPro®) was added to detach the cells. RNA was extracted using RNeasy Mini Kit (Qiagen) according to manufacturer’s specifications. RNA quantity and quality was assessed using NanoDrop A260/A280 OD readings. All RNA samples were quality tested to have readings of 260/280 absorbance between 1.8–2.0 and 260/230 absorbance of more than 1.0 (Nanodrop 2000, ThermoFisher Scientific Inc). Reverse transcription was carried using SuperScript® VILO™ cDNA Synthesis Kit (Invitrogen) that includes reverse transcriptase enzyme (SuperScript® III-RT), random primers and a recombinant ribonuclease inhibitor.

#### Real Time PCR

Quantitative real-time PCR was performed in ViiA™ 7 Real-Time PCR System (Applied Biosystems™) using SYBR Green PCR Master Mix reagent (Applied Biosystems™) and specifically designed primers (Supplementary file 1). Specific PCR products were detected with the fluorescent double-stranded DNA binding dye, SYBR Green. qRT-PCR amplification was performed in triplicates for each sample and the results were replicated in four independent experiments. Gel electrophoresis and melting curve analyses were conducted to validate PCR product sizes and the absence of nonspecific bands. The expression level of each gene was normalised against β-actin using the comparative CT method^74^.

#### ELISA assay

Cell culture supernatant from the SMF stimulated and Control (non-stimulated) group were collected every 48 hours and centrifuged at 2000g to remove cell debris. The samples were stored at −80 °C. The levels of Neurotrophin- BDNF and NT3 were quantified using an antigen capture enzyme-linked immunosorbent assay (ELISA) (Abcam) according to manufacturer’s protocol.

#### Calcium Imaging

OPCs were cultured in four well plates (IbidiR Inc.) slides and exposed to SMF for 2 weeks (2 hours/day). Calcium imaging was performed using Fluo-4 Calcium Imaging Kit (Molecular Probes), according to the manufacturer’s protocol. Briefly, OPCs were loaded with live cell imaging buffer (DMEM without Phenol Red (Invitrogen) supplemented with B27 (Gibco™), Human Insulin (Gibco™)) containing Fluo-4AM and Powerload Concentrate. The OPCs were incubated at 37 °C for 15 minutes followed by incubation for 15 minutes at room temperature. The cells were washed with PBS and suspended in the live cell imaging solution. For experiments with L-type calcium blocker Nifedipine, 10uM of Nifedipine (Sigma) was added to the cells and they were incubated for 15 minutes. All the calcium imaging experiments were performed using time lapse imaging (2 minutes, 5 second intervals) on a Ziess Confocal Microscope. Calcium influx was recorded by fluorescence emission at 488 nm after stimulation with 15 mM KCL (added at 30 seconds). Data was analyzed using FiJi software. The background fluorescence was subtracted using the ‘rolling-ball’ feature in ImageJ. Seven ROI’s surrounding the cell was selected to provide average background intensity. The fluorescent amplitude changes (ΔF_amplitude_) is defined as (F_t_ − F_o_)/F_o_) where F_t_ is the peak background-subtracted average pixel intensity at time t in a ROI divided by the background-subtracted baseline level (F_o_) in the same ROI. The results were expressed as mean amplitude ± SEM (n = 12 cells, 4 trials). Statistical analysis was performed using One-way ANOVA and Tukey’s multiple comparisons test. Representative video for calcium imaging experiments is provided in Supplementary Videos V1–V4.

### Compartmentalized Microfluidic fabrication and culture system

#### Microfluidic chamber fabrication

Devices for the compartmentalized culture consisted of two rectangular compartments (3.5 mm × 5.5 mm, 6 mm high) combined via parallel microchannel (500 μm long; 10 μm wide; 3 μm high; 35 μm spacing) as illustrated in Fig. 6a. The devices were fabricated through a two-step photolithography followed by a soft lithography with Polydimethylsiloxane (PDMS) as described earlier^75^. Briefly, a 3 μm layer of SU-8 2002 (MicroChem) was spun and baked on silicon wafer (University Wafer) before defining microchannels *via* exposure to UV light through a high resolution DPI transparency (Cad/Art) and baking. This process was repeated with SU-8 3050 (MicroChem) to create fluidic reservoirs at the microchannels ends. The patterned wafer served as master mold for casting PDMS (10:1 base to curing agent ratio; Dow Corning Sylgard 184 Silicone Elastomer) pads. The compartments were punched out at the reservoirs area using reshaped biopsy puncher (Ø = 6 mm; Ted Pella). The pads were then permanently bonded to glass coverslips (22 × 40 mm, no. 1 thickness; Menzel Gläser) *via* oxygen plasma. Devices were sterilized by 20 min long autoclave treatment (120 °C, 0.1 MPa), and incubated overnight at 4 °C with 100 µg mL^−1^ Poly-D-Lysine Hydrobromide (PDL; Sigma Aldrich) and 5 µg mL^−1^ Laminin (Invitrogen) in 10 mM Dulbecco’s Phosphate Buffer Solution (DPBS; Lonza). Prior to cell seeding the compartments were rinsed four times with DPBS and once with Neurobasal medium supplemented with 1% P/S and 5% FBS. Cells were plated by emptying both compartments and adding 5 μl of cell suspension (1 × 10^7^ cells mL^−1^) at the side of the somatic compartment adjacent to the microchannels. Cells were allowed to attach for 5 min and both compartments were filled with the culture medium. Devices were placed in Petri dishes containing sterile cotton ball in water to minimize media evaporation during incubation (37 °C, 5% CO_2_). Cultures were maintained by half medium exchange every 3 days. GlutaMAX-1 concentration was reduced to 0.5% after 3 days *in vitro* (DIV); FudR and NGF were excluded from the medium after 6 and 10 DIV, respectively.

#### DRG extraction and plating

All experimental procedures were performed in accordance and approved by the Institutional Animal Care and Use Committee (IACUC) of National University of Singapore. Dorsal root ganglion (DRG) neurons were obtained from embryonic day 15 (E15) Sprague-Dawley rat embryos^76^. The dissection was performed in Leibovitz’s-15 medium (L-15; Gibco) with 1% Penicilin/Streptomycin (P/S; Gibco). Collected tissue was cut into smaller pieces and incubated with 0.125% Trypsin-EDTA (Gibco) at 37 °C. After 30 min the trypsinization was blocked by adding 2.5% Fetal Bovine Serum (FBS; Gibco). The cells were washed twice with L-15 + P/S, mechanically dissociated by trituration, collected through centrifugation and resuspended in the culture medium: Neurobasal supplemented with 2% B27, 1% GlutaMAX-1 (all Gibco), 20 ng mL^−1^ Nerve Growth Factor (NGF; R&D Systems) and 13 µg mL^−1^ Flurodeoxyuridin (FudR; Sigma Aldrich).

#### Micro-fluidic co-culture

The differentiated OLs (Day 14, for both stimulated and control groups) were added to the axonal chamber at a cell density of 10 cells/µl in 100 µl of OL differentiation medium. The co-culture was maintained for 1 week at the end of which the cells were fixed for immunostaining. The samples were imaged using Zeiss LSM 880 confocal microscopy instrument. Image J (Fiji) was used for image analysis and quantification of immunofluorescence samples from 10 randomly chosen fields. For assessment of myelination, 20 slices of Z-Stack images were acquired at an interval of 1μm and analyzed using the Zeiss Zen Lite 2.3 analysis software.

#### Statistical analysis

All experiments were repeated independently in quadruplicates (n = 4), unless stated. All results are expressed as mean ± SEM. Unpaired student t-tests was used to analyse the statistical significance of the result (p < 0.05*, p < 0.01**) of all results except the calcium imaging analysis. One-way ANOVA and Tukey’s multiple comparisons test was used to analyse the calcium analysis results.

#### Data Availability

The datasets generated and/or analyzed during the current study are available from the corresponding author on reasonable request.

## Electronic supplementary material

Supplementary Video 1

Supplementary Video 2

Supplementary Video 3

Supplementary Video 4
